# Bioinformatics drives discovery in Biomedicine

**DOI:** 10.6026/97320630016013

**Published:** 2020-01-01

**Authors:** Ravindra Gujar, Bharat Panwar, Sandeep Kumar Dhanda

**Affiliations:** 1 Division of Vaccine Discovery, La Jolla Institute for Immunology, CA 92037, USA

**Keywords:** Bioinformatics, multi-omics, big data, opportunities and biomedicine

## Abstract

Bioinformatics has evolved from providing basic solutions, such as sequence alignment, structure predictions, and phylogenetic analysis to an independent data-driven field. The unprecedented growth of genomic technologies and the enormous data have opened an avenue for bioinformaticians (Bioinformatics professionals) never been seen before in the history of mankind. The novel opportunity also requires creative solutions that need skills to deal with noisy, unstructured information to offer valuable biological insights. Currently, we are seeing only the tip of an iceberg and the future will revolve around big data sets in all forms of biological research. The emerging challenge is to unfold the hidden iceberg of data.

## Description:

The human genome sequencing project was the turning point for bioinformatics and it continues to expand like a black hole with the help of big consortia such as ENCODE, GTEx, NCI-GDC, human proteome map, CCLE, human microbiome, and the human cell atlas project. Initially, bioinformaticians were considered as assistants and were limited to work on a proposed hypothesis, which later followed by some wet-lab experiments. However, now we are entering a new arena of hypothesis-free and discovery-based solutions relying on the big data and fortunately, a huge amount of data is available in the public domain, therefore hypothesis built on one cohort/dataset could be tested on the other cohorts/datasets as well.

**Figure 1:**
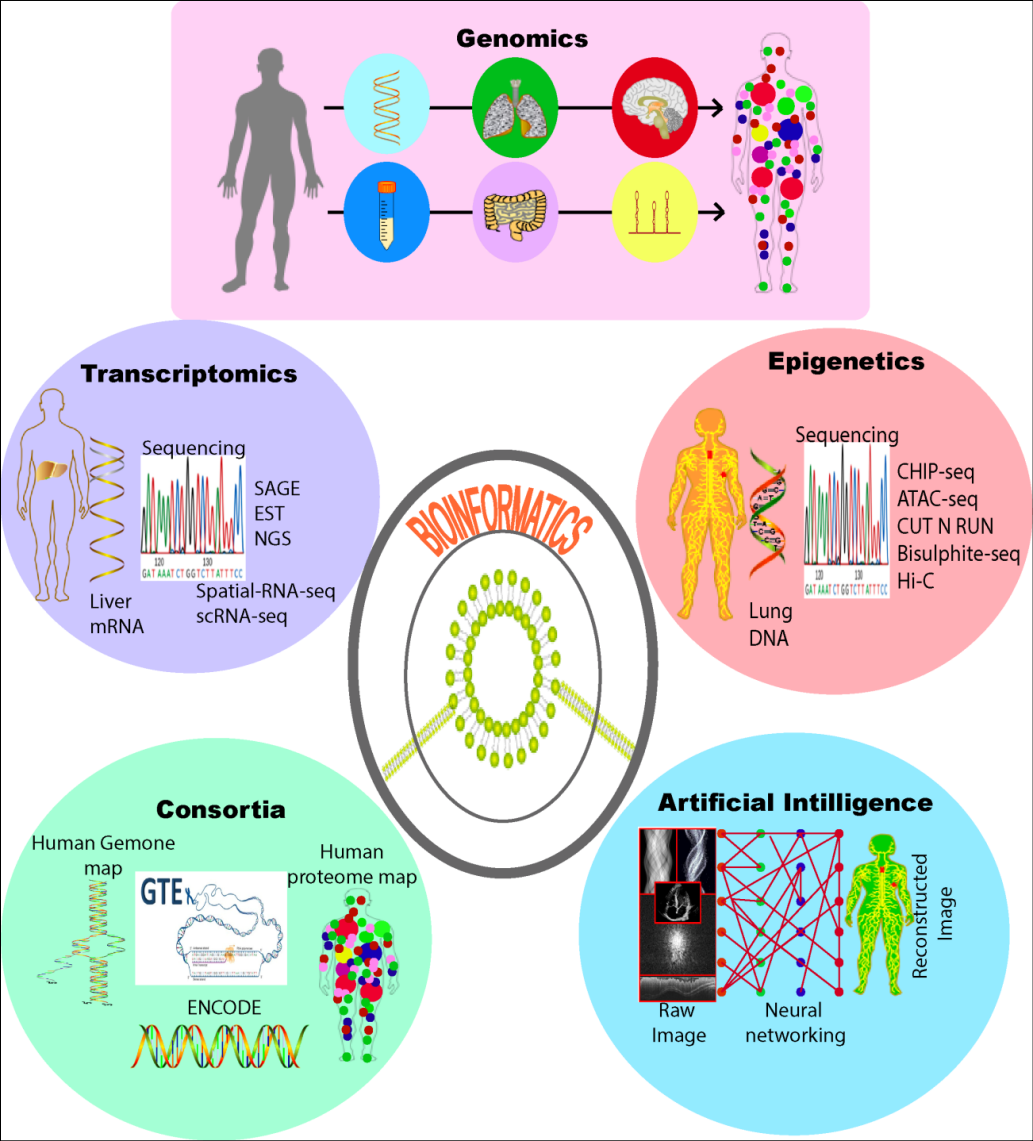
Schematic diagram to visualize the different arms of Bioinformatics. The integration of this omics information is propelling Bioinformatics to drive discovery in biomedical research.

Nowadays, artificial intelligence (AI) is impacting every aspect of human life and also reformed the bioinformatics field. For example, the Nature methods journal has recently published an issue in December 2019 to acknowledge the progress of deep learning models where these models have shown significant improvement over the traditional microscopy for detecting objects. Similarly, the AI-based protein-folding algorithm, Alpha Fold, outperformed existing methods at CASP-13 competition for predicting 3D protein structures from given amino acid sequences [1]. This technology further extended to help radiologists for analyzing mammographs, and also precisely identifying the hairline fractures. Although these prediction models are misrepresented as a black-box, it is essential to foresee futuristic interpretations coming from these AI outputs. Furthermore, the role of bioinformatics is revolutionizing healthcare by providing AI systems to assist physicians with reliable predictions that can never be overruled. These mathematical algorithms do snorkelling and diving with data, in a way the human mind or eye cannot do, detecting features that might be impossible to catch. Bioinformaticians are constantly developing these deep learning algorithms to delineate complex cellular images, powerful drug discovery, genomic new linkage. Thus, it's important to integrate different kinds of datasets (genomics and imaging to electronic medical records) in a single-chain to get a better insight (Figure 1).

Big technology companies are expanding the potentials of healthcare data and filling the required skill gap to efficiently manage and utilize digital data. Google has taken one step further and already showed its supremacy in quantum computing to meet with the growing power of data science [2]. Many market-players are developing devices/trackers to electronically record every bit of possible information from patients and healthy individuals. For example, the Apple smart watch's heart pulse monitor can detect real-time arterial fibrillation. The perspective of biology is like a metamorphosis of a butterfly, constantly changing and moulding into new forms. This trend of change is very evident, as many big technology companies are developing new algorithms, which will have imprints in defining the future of human health. Therefore, bioinformaticians are acquiring data science skills, that have been ranked as the sexiest job in the 21st century [3].

In clinical diagnostics, the paradigm has already shifted from a time-consuming culture-based classic microbiology approach to the rapid detection of thousands of pathogens using metagenomics approaches [4]. Newly emerging services are providing insight into the most suitable therapy available for an individual based on their genetic mutations. Additionally, existing ancestry related companies are now expanding their services toward genetic insight for wellness and AI-powered surgical robotics will change the medicine drastically with upcoming 5G technology. There are many government initiatives for large scale sequencing such as the UK has recently announced to sequence the whole genome for every newborn. It will empower the government to develop infrastructure for personalized medicine and will also give a better understanding of policy-making in healthcare.

In these last two decades, many institutions have accepted bioinformatics as a full-fledged discipline and now they are diversifying it further through setting up the departments of AI, data science and deep learning. The field has tremendous opportunities for the tech-savvy millennial and data enthusiast. New opportunities come with some risks and bioinformatics is no exception to it. This big data is also coming with noise and we have the challenge to find the needle in a haystack. The field has emerged from the crowd-sourced public/customer data but we believe that there are threats of data privacy and sanity. Although, block-chain based technology has already been extended to share peer-to-peer and encrypted genetic information. We are hopeful that bioinformaticians will accelerate the ride and will find a path forward for future medicines.
